# Early Tendon Transfer in Diabetic Hand Infection

**DOI:** 10.7759/cureus.83189

**Published:** 2025-04-29

**Authors:** Sushmi Ilavarasan, Manimaran R, Koppolu Kanchana, T Raghupathy

**Affiliations:** 1 General Surgery, Sree Balaji Medical College and Hospital, Chennai, IND; 2 Plastic Surgery, Sree Balaji Medical College and Hospital, Chennai, IND; 3 General Surgery, Bharath Institute of Higher Education and Research, Chennai, IND

**Keywords:** compartment syndrome, diabetes mellitus, disability, hand infections, multidisciplinary approach, tendon transfer

## Abstract

Hand infections in diabetic patients are a serious concern, as they can lead to significant functional impairment and, if not promptly treated, may result in extensive tissue destruction or even sepsis. Unlike diabetic foot infections, hand infections are less understood and often present unique challenges in management. Diabetic patients are at an increased risk due to factors such as neuropathy, impaired immune function, and poor wound healing. These infections, if neglected, can result in permanent disability. This report presents a rare case of a diabetic hand infection that was complicated by compartment syndrome, a condition that further exacerbates the risk of tissue damage. Due to the nature of the infection and the rapid progression of compartment syndrome, immediate surgical intervention was necessary. Early tendon transfer, a technique not commonly used in such infections, was employed to restore function and prevent further damage. The case highlights the importance of early recognition and aggressive management of diabetic hand infections. Prompt surgical intervention, including tendon transfer, can play a critical role in preventing long-term disability. This case contributes to the limited body of literature on diabetic hand infections, emphasizing the need for tailored treatment approaches to address the specific complications that arise in such cases.

## Introduction

Diabetic patients are particularly susceptible to upper limb infections due to their compromised immune systems, which predispose them to severe soft tissue complications if not aggressively treated [1]. Even minor arm injuries in diabetics can escalate into necrotizing fasciitis (NF), a rapidly progressing infection associated with extensive tissue necrosis, tendon involvement, and systemic toxicity. Early and aggressive surgical debridement, often involving multiple procedures, is critical, as delays beyond 24 hours significantly increase mortality [2]. In a retrospective study of 33 diabetic patients with upper extremity NF, 36.4% required amputation and 21.2% died during treatment, with common complications including septic shock and acute renal insufficiency [3]. Although rare, compartment syndrome secondary to hand infection demands immediate surgical intervention due to the risk of irreversible muscle and nerve damage [4]. In such cases, early tendon transfer, such as reconstruction of the flexor pollicis longus using the palmaris longus tendon, has demonstrated favorable outcomes and facilitates early rehabilitation [5]. These findings highlight the importance of early recognition, prompt surgical management, and timely reconstructive procedures in diabetic patients with upper limb infections to preserve function and reduce morbidity and mortality.

## Case presentation

**Figure 1:**
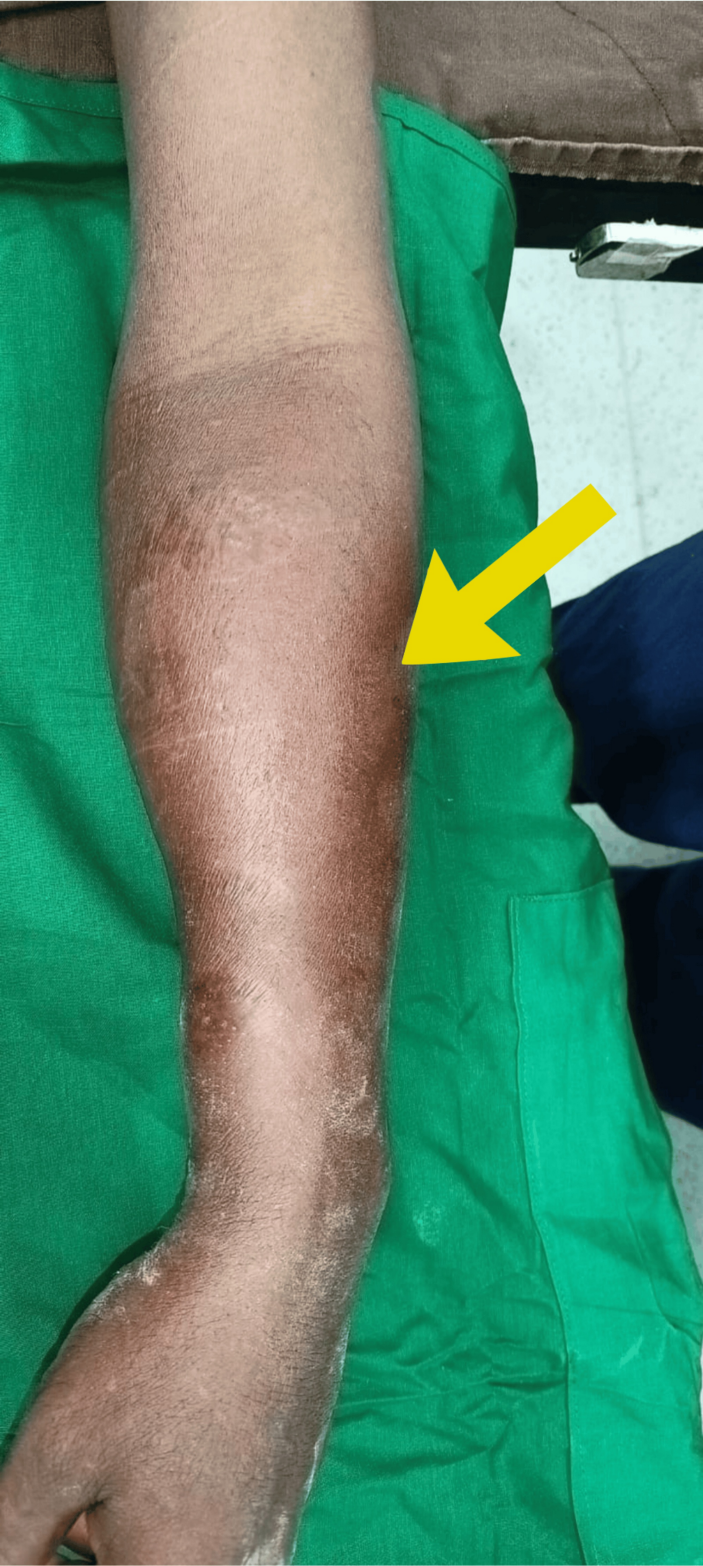
Swelling in the left forearm

A 46-year-old male was brought to the casualty with pain and swelling over the left forearm for a duration of one week. The patient had an alleged history of trauma by forceful pull of the left arm by another person. The patient also had a history of diabetes mellitus for one year, not on regular medications. On examination of the left forearm, a diffuse tender swelling was present over the volar aspect of the left forearm extending from the joint line of the elbow to the mid-forearm (Figure 1). Redness was present along with tenderness and restriction of movements (supination and pronation). The left radial and ulnar arteries were feeble compared to the right side. The brachial pulse was not felt. The flexor digitorum profundus of the index finger and the flexor digitorum longus of the thumb were not moving.

Ultrasound of the left forearm swelling showed a heterogeneous collection with multiple septations and echogenic debris noted in the ventral aspect of the forearm, extending just below the elbow to the wrist joint. Color Doppler shows minimal vascularity to the forearm and hand. An abscess is suspected. The underlying bones and muscles appear normal. MRI/CT of the hand has not been done due to the emergency toxic state. Hence, the patient was taken up for emergency fasciotomy and debridement without any delay.

Complete surgical approach

**Figure 2:**
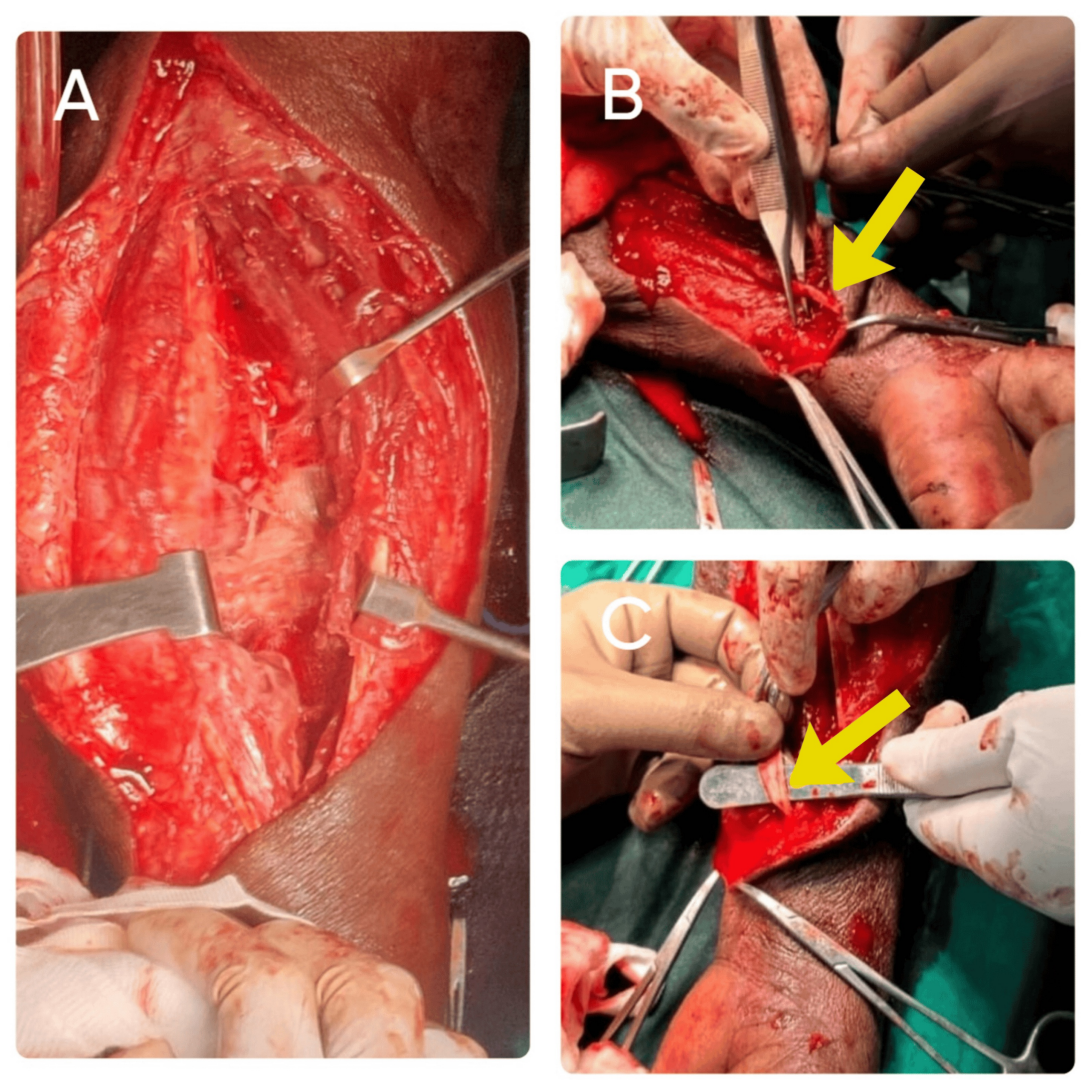
Wound debridement of the flexor pollicis longus (A) The patient was taken up for emergency fasciotomy and wound debridement. Fasciotomy was performed to relieve the compartment pressure. (B) An extensive debridement of the necrotic flexor pollicis longus and flexor digitorum superficialis muscles. (C) Necrotic flexor pollicis longus.

**Figure 3:**
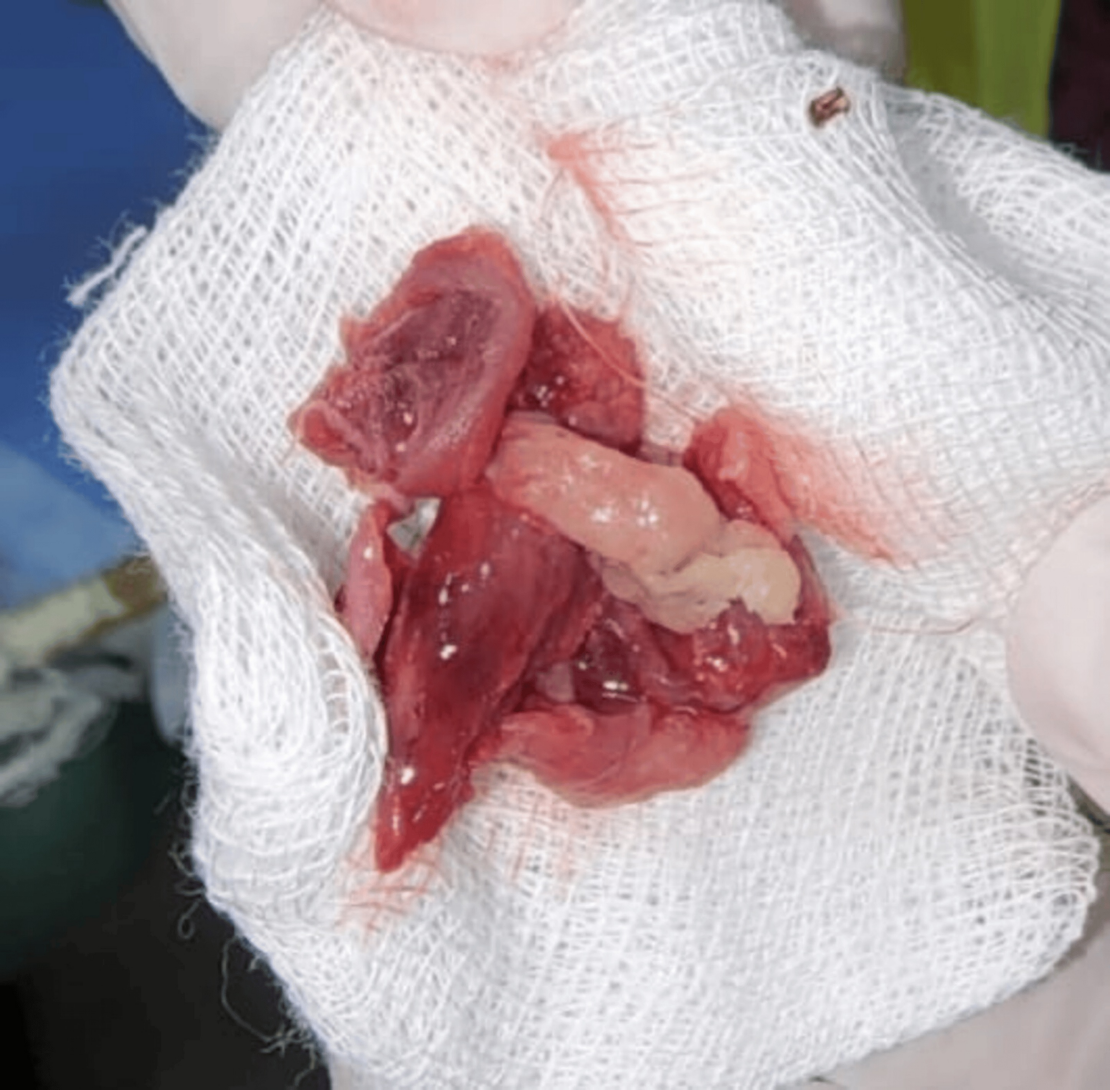
Excised part of the flexor pollicis longus tendon

**Figure 4:**
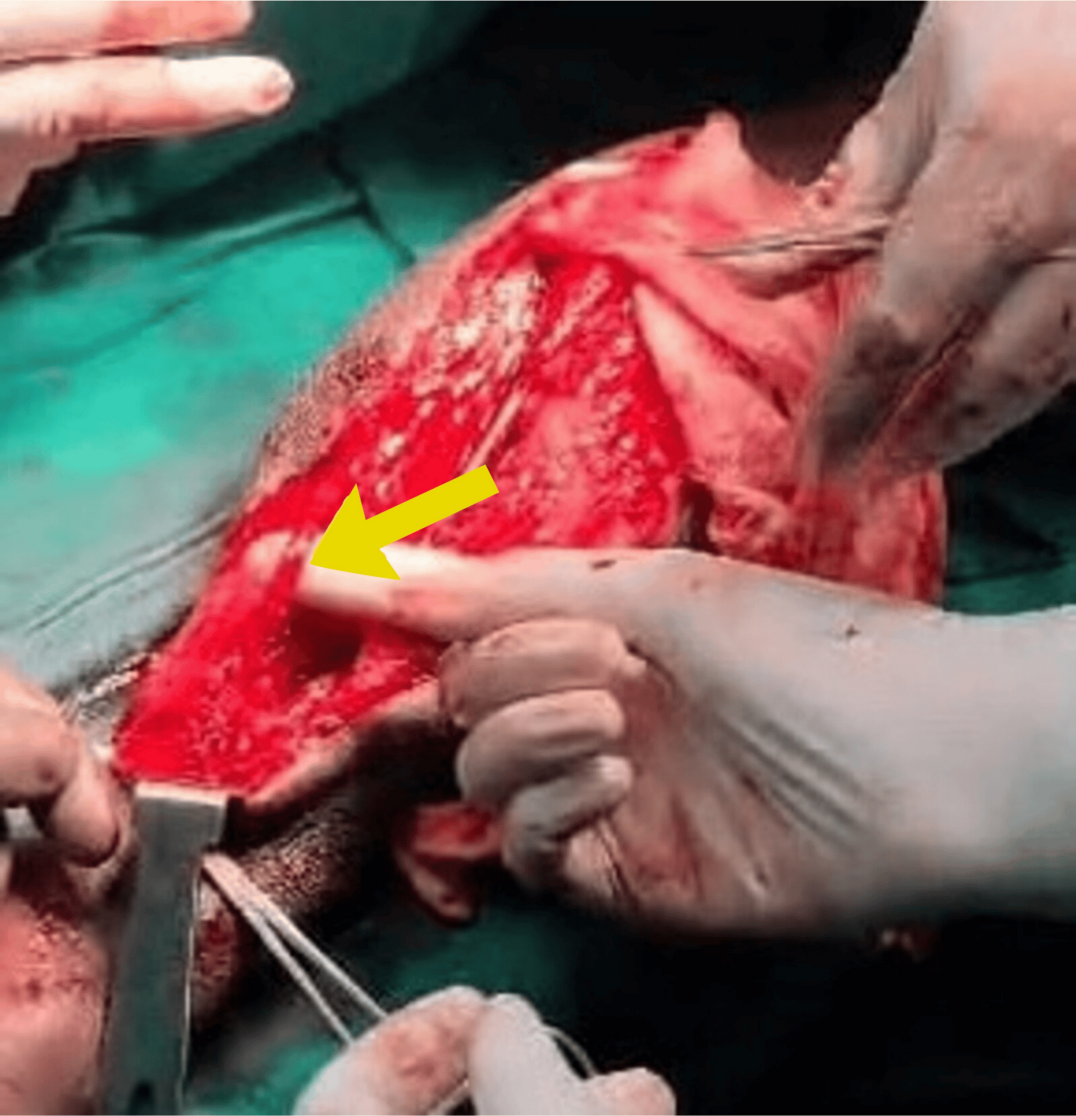
Tendon transfer of the palmaris longus to the flexor pollicis longus tendon The wound was left open, and regular dressings were done. After 20 days of wound stabilization, the palmaris longus tendon was transferred to replace the necrotic flexor pollicis longus tendon for restoration of thumb flexion. This early reconstructive step was chosen to prevent long-term disability and optimize functional restoration.

**Figure 5:**
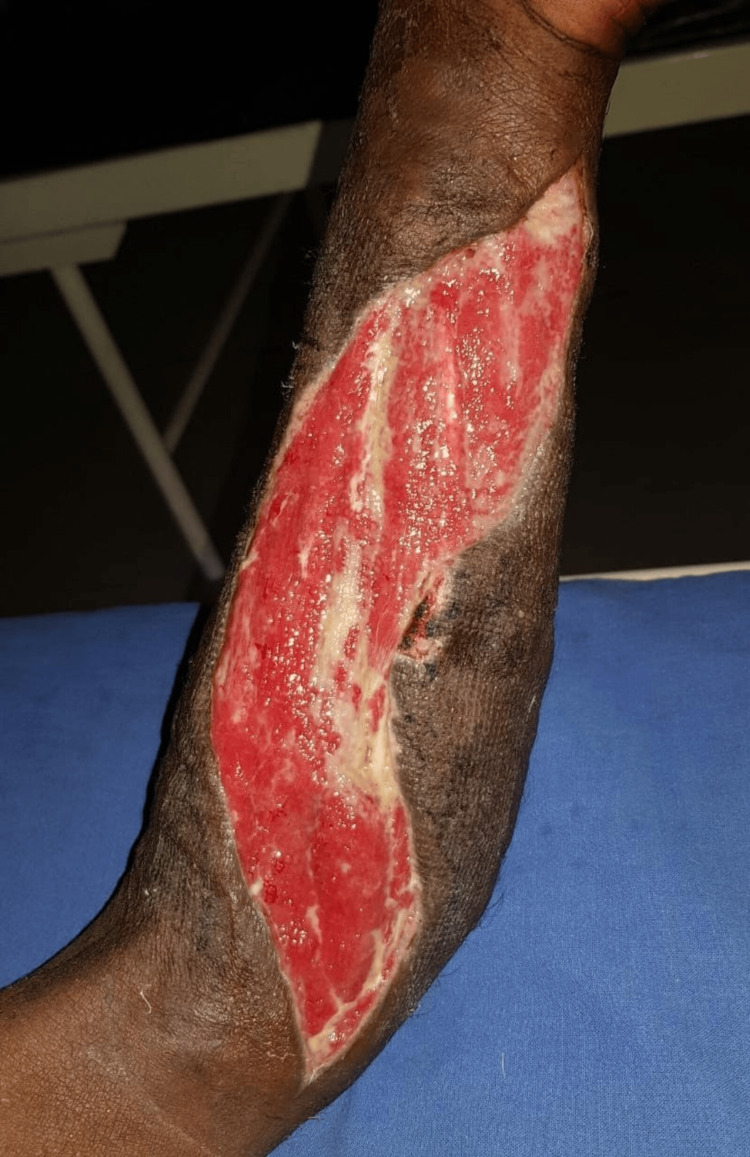
Raw area over the left forearm

**Figure 6:**
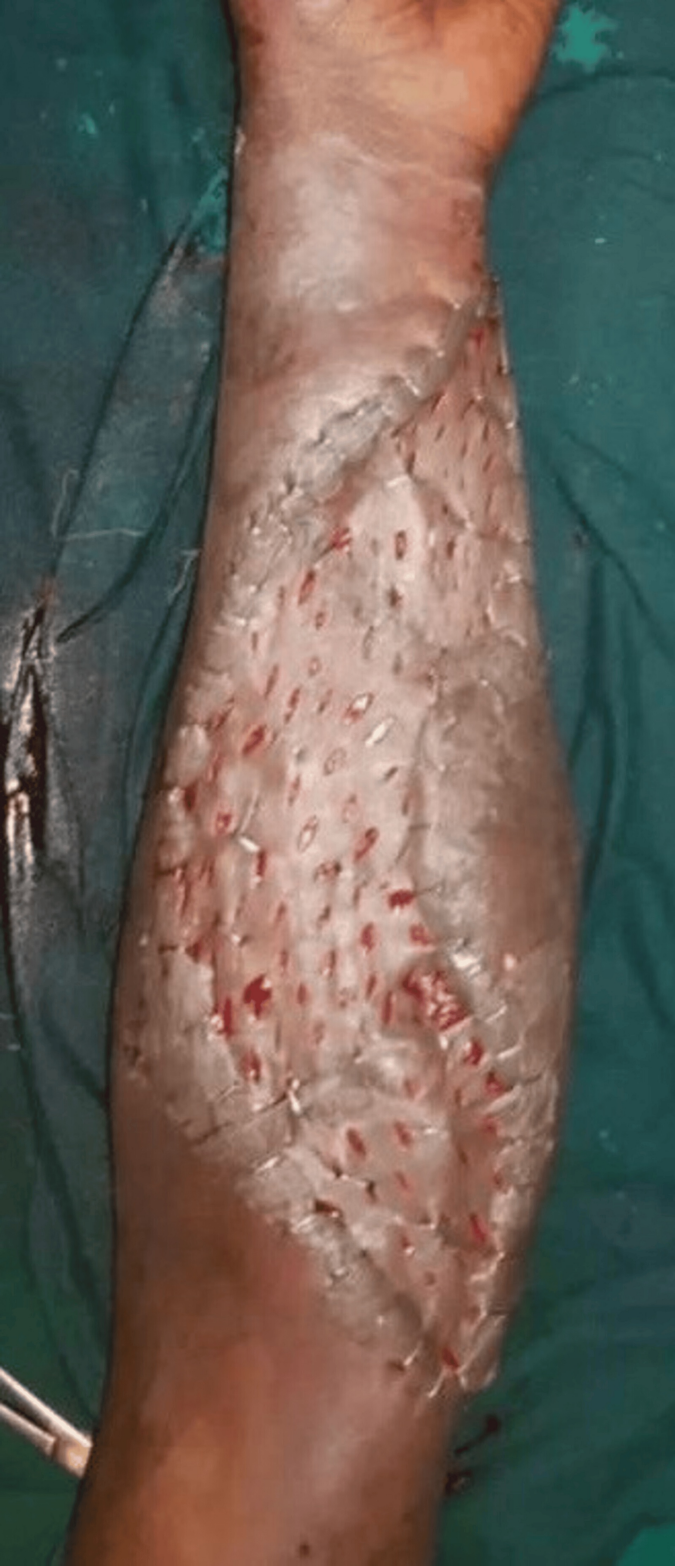
Split skin graft A split skin graft was placed after four weeks to cover the residual raw area to promote healing. The patient regained functional movement of the thumb within six weeks. Grip strength improved at the follow-up of 12 weeks. Healing progressed well with no evidence of graft loss or secondary deformity. Postoperative physiotherapy improved tendon glide and range of motion.

Emergency fasciotomy was performed without delay to decompress the affected muscle compartments and prevent further ischemic damage to the muscles and nerves (Figure 2). The volar (anterior) compartment was the primary site involved. The skin and underlying fascia were incised longitudinally to open the compartment and reduce pressure. Upon opening, necrotic tissue was found, particularly involving the flexor pollicis longus and flexor digitorum superficialis muscles (Figure 3). These were surgically debrided, meaning all dead and infected tissue was excised to prevent further spread of infection and to promote healing. The wound was left open post-procedure, and regular dressings were applied during the healing phase to monitor the wound and prevent further infection. Stabilization of the wound was done for approximately 20 days. As the wound was healthy and the distal tendon was adequate with distal skin cover, an intraoperative decision was made to transfer the palmaris longus tendon to the flexor pollicis longus, as delayed repair may not be possible if the distal tendon desiccates (Figure 4). The palmaris longus tendon was harvested from the same limb. The distal end of the necrotic flexor pollicis longus was debrided and prepared. The palmaris longus tendon was tunneled through the soft tissue and sutured to the distal stump of the flexor pollicis longus tendon. Proper tensioning was ensured so that the transferred tendon could reproduce the normal flexion arc of the thumb. Intraoperative assessment was likely done to verify the range of motion (ROM) and thumb flexion before closure. The patient had a residual raw area over the volar forearm following fasciotomy, debridement, and tendon transfer (Figure 5). After ensuring the infection was resolved and granulation tissue was healthy, a split skin graft (SSG) was performed four weeks postoperatively to promote definitive wound closure and enhance healing (Figure 6). Before grafting, the raw area was managed with regular dressings to ensure that the infection was cleared, healthy granulation tissue had formed, and there was no necrotic tissue or ongoing exudate. The donor site was taken from the thigh. The graft was trimmed to match the shape of the wound. It was then placed over the raw area and secured using staples around the edges. A non-adherent dressing was done over the graft. The donor area was dressed separately.

The postoperative course for the patient was progressive and favorable, beginning with meticulous wound care following the initial emergency fasciotomy and debridement, during which the wound was left open and managed with regular dressings to control infection and promote granulation. After 20 days, once the infection had subsided and the wound bed was stable, a tendon transfer was performed using the palmaris longus tendon to restore thumb flexion. The transferred tendon was monitored for viability, and the patient was kept under strict glycemic control and antibiotic coverage to prevent secondary infection. Four weeks later, a split skin graft (SSG) was applied over the residual raw area to ensure complete wound coverage and enhance healing. Postoperatively, physiotherapy was initiated in a phased manner, starting with passive range of motion exercises followed by active mobilization to improve tendon glide and prevent joint stiffness. The patient regained functional thumb movement within six weeks, and by 12 weeks, the patient demonstrated significant improvement in grip strength and hand utility, with no signs of graft loss, infection, or deformity, marking a successful recovery aided by a multidisciplinary approach.

Medical management

Empirical broad-spectrum antibiotics were initiated immediately due to the acute toxic presentation and suspected polymicrobial infection common in diabetic hand infections. Intravenous fluids were administered to maintain hydration and support renal perfusion, especially in the setting of systemic toxicity or sepsis risk. A third-generation cephalosporin, such as injection ceftriaxone, was started. The total antibiotic course likely lasted 2-4 weeks, tailored to clinical response and wound healing. For pain management, injection paracetamol was started and adjusted based on pain severity. The patient was started on insulin since he had a history of diabetes mellitus, but was not on regular medication, which significantly increased the risk of infection and impaired healing. Regular dressings were done and assessed for healthy granulation, infection signs, or necrosis.

Rehabilitation module

In weeks 0-3, immobilization was started: immobilization in a thumb spica splint with the wrist in 20°-30° flexion and thumb in moderate flexion and opposition to avoid tension on the repaired tendon, elevation of the limb above heart level to reduce edema, gentle passive ROM exercises for unaffected joints (shoulder, elbow, and fingers, except thumb) to avoid stiffness, and regular monitoring of the graft site and donor site for signs of infection or detachment. In weeks 3-6, early mobilization was started: passive thumb flexion and extension exercises under supervision and active thumb flexion against resistance. Splinting was continued except during therapy sessions. Scar management techniques (gentle massage and silicone gel sheets) were initiated over healed areas. In weeks 6-8, controlled active action was started: ROM exercises for thumb flexion and extension. Light functional tasks, such as holding a soft sponge or ball, were started. In weeks 8-12, strengthening and functional use were started. Progressive grip and pinch strengthening exercises were done. Functional training for activities of daily living was done. After 12 weeks, full active and passive ROM exercises were started, as well as strengthening routines tailored to the patient's occupational needs. Any signs of tendon tightness or joint contracture were monitored. Lifestyle modification and strict diabetes control were advised to prevent recurrence. The patient achieved functional thumb movement by six weeks and improved grip strength by 12 weeks, with no evidence of graft loss, contracture, or deformity.

## Discussion

Hand infections in diabetic patients present a unique and serious clinical challenge [6]. Due to the metabolic and vascular complications inherent in diabetes mellitus, these infections often have an aggressive clinical course. The impaired immune response, characterized by reduced neutrophil chemotaxis, phagocytosis, and intracellular killing, contributes significantly to delayed clearance of pathogens [7]. In addition, diabetic microangiopathy compromises tissue perfusion, further impairing the host's ability to mount an effective immune response and deliver antibiotics to infected tissues. Moreover, hyperglycemia fosters a favorable environment for bacterial proliferation and disrupts normal wound healing.

The most frequently implicated organisms in diabetic hand infections include *Staphylococcus aureus*, including methicillin-resistant *Staphylococcus aureus*, and *Streptococcus pyogenes*, although polymicrobial infections are also common, especially in more severe or chronic cases [8]. These infections often require prompt surgical debridement, broad-spectrum antibiotic therapy, and close monitoring due to their rapid progression and the potential for tissue necrosis, tendon exposure, and joint involvement [9].

In the present case, following initial debridement, the patient suffered loss of the flexor pollicis longus muscle, which resulted in loss of thumb flexion. This is particularly significant as the thumb accounts for approximately 40%-57% of overall hand function, primarily due to its role in pinch and grip. Thumb dysfunction drastically reduces hand utility and the patient's ability to perform daily activities, reinforcing the need for timely and effective reconstructive interventions. Following a second debridement, the wound condition improved, with adequate distal skin coverage. Based on intraoperative findings, an on-table decision was made to transfer the palmaris longus tendon to the distal stump of the flexor pollicis longus as a salvage procedure. Tendon transfer surgeries are traditionally reserved for patients after complete wound healing and soft tissue recovery; however, in selected cases with favorable local conditions and to prevent contractures and prolonged immobilization, early functional reconstruction can be considered. The palmaris longus is a well-established donor tendon for functional reconstruction due to its superficial location, expendability, and adequate length for transfer [10]. Its use in flexor pollicis longus tendon reconstruction has shown satisfactory outcomes in terms of grip strength, pinch function, and early return to activity [11]. Early tendon transfer in this context is justified by the need to preserve thumb mobility, shorten rehabilitation duration, and improve overall hand strength, ultimately leading to better patient outcomes [12].

The use of the palmaris longus tendon transfer for flexor pollicis longus reconstruction in diabetic hand infections offers several advantages, particularly its superficial location, expendability, and adequate length, making it ideal for early functional restoration in patients with good local wound conditions. Compared to other techniques, such as flexor digitorum superficialis transfer, which provides stronger flexion but sacrifices finger function and requires more complex dissection, the palmaris longus transfer is less invasive with minimal donor morbidity. Brachioradialis transfer, another option, offers robust power but may impair elbow function and entails longer rehabilitation [13]. Free tendon grafts (e.g., plantaris) are suitable when motor units are intact, but they lack active function without additional transfers. In contrast, thumb arthrodesis provides a stable thumb for pinch but eliminates mobility, making it more suitable for low-demand patients or those unfit for reconstruction. Thus, early palmaris longus transfer strikes a balance between simplicity, functional recovery, and minimal morbidity, especially crucial in diabetics where rapid intervention and rehabilitation are key to preserving hand utility.

## Conclusions

Diabetic hand infections can be severe and disabling, requiring early surgical intervention. Early tendon transfer, combined with fasciotomy and appropriate infection control, can significantly improve functional outcomes. This case highlights the importance of timely reconstructive procedures in diabetic hand infections, emphasizing the need for a multidisciplinary approach to optimize patient recovery. Hence, the hand function of this patient has been restored with the timely use of the salvage procedure.
